# Acute psychosis following initiation of ruxolitinib in post-polycythaemia vera myelofibrosis case report

**DOI:** 10.1007/s00277-026-06764-0

**Published:** 2026-01-23

**Authors:** Louise Jade Potter, Sachin Shetty, M. Mansour Ceesay

**Affiliations:** 1 Department of Haematological Medicine, King’s College Hospital NHS Foundation Trust, London, UK; 2 Kent and Medway NHS and Social Care Partnership Trust, Maidstone, UK

**Keywords:** Ruxolitinib, Polycythaemia vera, Myelofibrosis, Psychosis, Case report

## Abstract

Polycythaemia vera (PV) is a myeloproliferative neoplasm (MPN) which can progress to myelofibrosis (MF), a phenomenon termed post-PV MF. Ruxolitinib is a Janus activated kinase (JAK) inhibitor that targets JAK1/2 mutations and is approved for treatment of high-risk MF. This is a case report of a patient with post-PV MF who developed acute psychotic disorder five months after commencing ruxolitinib and whose mental status returned to baseline within one week of stopping ruxolitinib. Importantly, there was no history of mental health disorder and no identifiable triggers for his presentation. Ruxolitinib has not previously been implicated in the development of psychosis but is known to cross the blood-brain barrier and affect multiple cell signalling pathways within the central nervous system. Together, this makes an association between ruxolitinib and neuropsychiatric symptoms plausible, yet further understanding of causal mechanisms is warranted.

**Supplementary Information:**

The online version contains supplementary material available at 10.1007/s00277-026-06764-0.

## Introduction

Myeloproliferative neoplasms (MPN) encompass a group of clonal haematological malignancies including chronic myeloid leukaemia, polycythaemia vera (PV), essential thrombocythemia (ET) and myelofibrosis (MF) [1], which arise from pluripotent haematopoietic stem cells. PV and ET are characterised by progressive hyperproliferation of erythrocytes or thrombocytes, and both can progress to MF, termed post-PV MF and post-ET MF, respectively. MF is characterised by bone marrow fibrosis resulting in progressive splenomegaly and pancytopenia. The World Health Organisation (WHO) grades MF from 0 to 3, with 0 representing healthy bone marrow and 3 indicating significant collagen fibrosis with osteosclerosis [2]. Janus activated kinase (JAK) 2 genetic mutations, V617F and exon 12, are recognised drivers of MPNs and ruxolitinib, a JAK1/2 inhibitor, is approved for treatment of high-risk MF. This is a case report of a patient with post-PV MF who developed acute psychosis following treatment with ruxolitinib.

## Case description

A Black African male with sickle cell trait initially presented at 40 years of age with a haemoglobin of 183 g/l, haematocrit 0.525 L/L, platelets of 602 × 10^9^/l with normal white blood counts and neutrophils. He was found to be JAK2 V617F positive with variable allele frequency (VAF) of 30% and undetectable serum erythropoietin of < 2.5 mU/mL. His bone marrow biopsy showed panmyelosis, with prominent erythroid and megakaryocytic proliferation and megakaryocytic clustering diagnostic of JAK2 V617F positive PV. He was treated with venesection and oral hydroxycarbamide of varying doses from 1000 mg to 2000 mg daily (adjusted based on haematocrit and platelet counts) with good response. After around 7 years, hydroxycarbamide was discontinued due to skin and nail hyperpigmentation. He was switched to oral anagrelide of varying doses from 0.5 mg to 1.0 mg twice daily (based on haematological response) and he remained on this with good control of his PV.

After approximately 10 years of anagrelide treatment, the patient developed a rising platelet count of 667 × 10^9^/l, indicating a loss of therapeutic response, although his spleen size remained normal at 11.7 cm on abdominal ultrasound. A repeat bone marrow biopsy demonstrated hypercellularity (> 90% cellularity) with panmyelosis and WHO grade 3 fibrosis, features consistent with post-PV MF. Our myeloid gene panel (see supplementary material) demonstrated persistence of the JAK2 V617F mutation (VAF 48%) and the presence of two ten-eleven translocation 2 (TET2) mutations, TET2 p.(Cys1193Tyr) (VAF 6%) and TET2 p.(lle830ThrfsTer15) (VAF 18%). Anagrelide was stopped and oral ruxolitinib was commenced at 15 mg twice daily for 3 months and then increased to 25 mg twice daily.

**Fig. 1:**
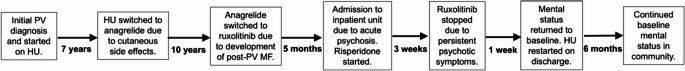
A timeline of key clinical events. PV = polycythaemia vera, HU = hydroxycarbamide, MF = myelofibrosis. Timeline not drawn to scale

After a total of five months of ruxolitinib treatment, at 60 years of age, the patient presented with insomnia, sudden onset of persecutory delusions and auditory hallucinations. Investigations including a head CT scan and serology screening for human immunodeficiency virus and syphilis were negative. Autoimmune screening for leucine-rich glioma-inactivated 1, N-methyl-D-aspartate receptor and contactin-associated protein-like 2 antibodies was negative. Therefore, an organic cause for his change in mental status was not detected. Following a full neuropsychiatric assessment by a consultant psychiatrist a diagnosis of acute psychotic syndrome was made, and the patient was admitted to hospital under section two of the UK Mental Health Act. Importantly there was no past or family history of psychiatric illness and, aside from unspecific work-related stress, there were no identified precipitating factors for the onset of psychosis. He did not smoke cigarettes or cannabis nor take any other illicit medications. He was treated with oral risperidone 2 mg daily but continued to experience florid psychotic symptoms. Ruxolitinib was the only recently started medication and around three weeks into the hospital admission, this was stopped, following haematology advice. Following this his mental status made rapid recovery and one week after the cessation of ruxolitinib he was discharged, following a total of four weeks as an inpatient. Hydroxycarbamide was restarted as an alternative treatment of his post-PV MF. He remains well for over six months and is regularly followed up by the community mental health and outpatient haematology teams. A timeline of the key clinical events is summarised in Fig. 1.

## Discussion

In this case report the temporal association between starting ruxolitinib, the onset of psychosis and the rapid resolution of symptoms upon cessation of the drug implies a possible association between ruxolitinib and acute psychotic syndrome. This is further supported by the absence of previous mental health disorder, precipitating factors and identifiable organic causality.

Ruxolitinib is known to cause haematological adverse effects including anaemia, thrombocytopenia and neutropenia, as well as non-haematological adverse effects such as fatigue, abdominal pain and diarrhoea. Ruxolitinib is reported to cause mild neurological adverse effects such as headache and dizziness. However, to our knowledge the association of ruxolitinib and acute psychotic disorder has not been previously reported. A single case report implicates ruxolitinib in the development of progressive multifocal leukoencephalopathy (PML) [3]. In this case the authors describe the development of progressive cognitive impairment, expressive dysphasia and gait disturbance ten weeks following initiation of ruxolitinib. Brain imaging and biopsy were diagnostic for PML, indicating infective pathology, unlike in our case where no organic pathology was found. Another single case report describes episodic hypoesthesia and weakness of the right arm and leg three weeks following initiation of ruxolitinib, which resolved on cessation of the drug [4]. Unlike our case report, no central nervous system (CNS) symptoms were described. Interestingly, a single case report associates tofacitinib, a JAK1/3 inhibitor with acute mania [5]. In this report, tofacitinib was initiated as treatment for acute severe ulcerative colitis. After three months of treatment, the patient presented with aggressive behaviour, excessive speech, agitation and sleep disturbance, which resolved two weeks after cessation of tofacitinib. The neuropsychiatric presentation, temporality of symptom development and rapid resolution following cessation of the JAK inhibitor described is comparable to our case report. However, unlike ruxolitinib, tofacitinib inhibits JAK3, and the inhibition of JAK3 phosphorylation is implicated in the mechanism of action for tricyclic antidepressants [6], which poses a plausible association with the mania described.

Ruxolitinib has the ability to cross the blood-brain barrier (BBB) [7] which has led to the investigation into its potential therapeutic effect in CNS malignancies [8] and neurodegenerative disorders [9]. Within the CNS, ruxolitinib has been found to regulate the Wingless/Integrated (WNT) signalling pathway, promoting cytotoxic and apoptotic effects of chemotherapeutic agents [8]. Moreover, the WNT signalling pathway has been implicated in the growth and differentiation of dopaminergic neurones [10]. Given that dopaminergic dysfunction plays a crucial role in psychosis pathophysiology, particularly that of the positive symptoms reported in our case, the WNT signalling pathway poses a potential mechanism for ruxolitinib to cause neuropsychiatric effects. In addition, ruxolitinib has been shown to attenuate CNS neuroinflammation through inhibition of the mitogen-activated protein kinase/nuclear factor-κB signalling pathway and reduced microglia and astrocyte activation [9]. Together, ruxolitinib’s ability to cross the BBB, effect on multiple cell signalling pathways and immunomodulation within the CNS make its association with neuropsychiatric symptoms plausible.

Although ruxolitinib reaches a steady-state plasma concentration by the second day of twice daily dosing [11], we report a five-month delay between treatment initiation and the development of neuropsychiatric symptoms. This is comparable to the three-month delay between the initiation of tofacitinib and development of mania previously reported [5]. Such delay is also seen in well-known adverse effects of ruxolitinib, including myelosuppression, which can emerge months after treatment initiation [12]. The delay between achieving a steady-state plasma concentration and the development of the neuropsychiatric phenotype described are possibly due to the cumulative alteration of cell signalling pathways and exhaustion of initial compensatory mechanisms.

A limitation of this case report is that the accurate identification of precipitating factors may be affected by reporting bias, resulting in possible aetiological factors being overlooked. Moreover, although investigative efforts were made to eliminate an organic cause, including a CT head, virology screening and autoantibodies, this was not exhaustive and additional investigations such as an MRI brain, lumbar puncture and toxicology screen may have helped established causality. However, given the absence of physical symptomatology and complete resolution of mental status on cessation of ruxolitinib, an organic cause is less likely. Ultimately, given the complex multifactorial aetiology of psychosis it is difficult to eliminate all confounding factors and establish direct causative mechanisms. Therefore, despite the strong temporal association between starting ruxolitinib, the onset of psychotic symptoms and the rapid resolution of symptoms on its cessation, these observations may be coincidental and better understanding of potential causal mechanisms is warranted.

## Conclusion

This is a case report of a patient with post-PV MF who developed acute psychotic disorder five months after commencing ruxolitinib and who’s mental status rapidly recovered on cessation of ruxolitinib. Importantly there was no history of mental health disorder and no identifiable triggers for his presentation. Ruxolitinib has not previously been implicated in the development of psychosis but is known to effect multiple cell signalling pathways in the CNS, making an association with neuropsychiatric symptoms plausible.

## Supplementary Information

Below is the link to the electronic supplementary material.

Supplementary Material 1

## Data Availability

No datasets were generated or analysed during the current study.

## References

[1] Tefferi A, Vardiman JW (2008) Classification and diagnosis of myeloproliferative neoplasms: the 2008 world health organization criteria and point-of-care diagnostic algorithms. Leukemia 22(1):14–22 17882280 10.1038/sj.leu.2404955

[2] Swerdlow SH, Campo E, Harris NL, Jaffe ES, Pileri SA, Stein H et al (2008) WHO classification of tumours of Haematopoietic and lymphoid tissues. International agency for research on cancer Lyon, France

[3] Wathes R, Moule S, Milojkovic D (2013) Progressive multifocal leukoencephalopathy associated with ruxolitinib. N Engl J Med 369(2):197–198 23841743 10.1056/NEJMc1302135

[4] Furia F, Canevini MP, Federici AB, Carraro MC (2020) Unexpected neurological symptoms of ruxolitinib: A case report. J Hematol 9(4):137–139 33224394 10.14740/jh642 PMC7665860

[5] Dalai DMK, Ingle DM, Singh DG, Pandey DV, Chauhan DS, Bairwa DY (2023) A case of Tofacitinib induced mania. Psychiatry Res Case Rep 2(1):100114

[6] Fukuyama T, Tschernig T, Qi Y, Volmer DA, Bäumer W (2015) Aggression behaviour induced by oral administration of the Janus-kinase inhibitor tofacitinib, but not oclacitinib, under stressful conditions. Eur J Pharmacol 764:278–282 26164790 10.1016/j.ejphar.2015.06.060

[7] Haile WB, Gavegnano C, Tao S, Jiang Y, Schinazi RF, Tyor WR (2016) The Janus kinase inhibitor ruxolitinib reduces HIV replication in human macrophages and ameliorates HIV encephalitis in a murine model. Neurobiol Dis 92(Pt B):137–143 26851503 10.1016/j.nbd.2016.02.007 PMC4907871

[8] Goker Bagca B, Ozates NP, Biray Avci C (2022) Ruxolitinib enhances cytotoxic and apoptotic effects of Temozolomide on glioblastoma cells by regulating WNT signaling pathway-related genes. Med Oncol 40(1):37 36460932 10.1007/s12032-022-01897-4

[9] Min J, Zheng H, Xia H, Tian X, Liang M, Zhang J et al (2024) Ruxolitinib attenuates microglial inflammatory response by inhibiting NF-κB/MAPK signaling pathway. Eur J Pharmacol 968:176403 38354846 10.1016/j.ejphar.2024.176403

[10] Castelo-Branco G, Arenas E (2006) Function of Wnts in dopaminergic neuron development. Neurodegenerative Dis 3(1–2):5–11 10.1159/000092086 16909030

[11] Shi JG, Chen X, McGee RF, Landman RR, Emm T, Lo Y et al (2011) The pharmacokinetics, pharmacodynamics, and safety of orally dosed INCB018424 phosphate in healthy volunteers. J Clin Pharmacol 51(12):1644–1654 21257798 10.1177/0091270010389469

[12] Srdan V, Ruben AM, Jason G, Richard SL, Vikas G, John FD et al (2013) Efficacy, safety and survival with ruxolitinib in patients with myelofibrosis: results of a median 2-year follow-up of COMFORT-I. Haematologica 98(12):1865–1871 24038026 10.3324/haematol.2013.092155 PMC3856961

