# Supplementary material for: Acute psychosis following initiation of ruxolitinib in post-polycythaemia vera myelofibrosis case report
Source: Ann Hematol. 2026 Jan 23;105(2):53. doi: 10.1007/s00277-026-06764-0 (PMC12827360; doi:10.1007/s00277-026-06764-0)
Supplement: Supplementary file 1 — Supplementary Material 1 [file 277_2026_6764_MOESM1_ESM.docx]

**Supplementary Material**

| **Gene Name** | **Gene Region** |
| --- | --- |
| ANKRD26 | All coding and 3' UTR |
| ASXL1 | Exon 12 |
| BCL2 | All coding |
| BCOR | All coding |
| CALR | Exon 9 |
| CBL | Exons 7+8+9 |
| CEBPA | All coding |
| CSF3R | Exons 13 to 18 |
| CUX1 | All coding |
| DDX41 | All coding |
| DNMT3A | All coding |
| ETV6 | All coding |
| EZH2 | All coding |
| FLT3 | Exons 14+15+20 |
| GATA1 | All coding |
| GATA2 | All coding |
| GNB1 | All coding |
| HRAS | Exons 2+3 |
| IDH1 | Exon 4 |
| IDH2 | Exon 4 |
| IKZF1 | Exons all coding |
| JAK2 | All coding |
| KIT | Exons 2, 8 to 11, 13+17 |
| KMT2A | All coding |
| KMT2C | All coding |
| KRAS | Exons 2+3 |
| MPL | All coding |
| NF1 | All coding |
| NFE2 | All coding |
| NPM1 | Exon 12 |
| NRAS | Exons 2+3 |
| PHF6 | All coding |
| PPM1D | All coding |
| PTPN11 | Exons 3+13 |
| RAD21 | All coding |
| RUNX1 | All coding |
| SETBP1 | Exon 4 |
| SF3B1 | (exons 12 to 16) |
| SH2B3 | All coding |
| SRSF2 | Exon 1 |
| STAG2 | All coding |
| STAT5B | All coding |
| TET2 | All coding |
| TP53 | All coding |
| U2AF1 | Exons 2+6 |
| UBA1 | All coding |
| WT1 | Exons 7+9 |
| ZRSR2 | All coding |

**Table S1**: Myeloid gene panel contents

Table outlining the name and the region of 48 genes that are sequenced by the myeloid gene panel.
